# Demarcation of Stable Subpopulations within the Pluripotent hESC Compartment

**DOI:** 10.1371/journal.pone.0057276

**Published:** 2013-02-21

**Authors:** Sonam Bhatia, Carlos Pilquil, Ivana Roth-Albin, Jonathan S. Draper

**Affiliations:** 1 McMaster Stem Cell and Cancer Research Institute, Michael G. DeGroote School of Medicine, McMaster University, Hamilton, Ontario, Canada; 2 Department of Pathology and Molecular Medicine, McMaster University, Hamilton, Ontario, Canada; 3 Department of Biochemistry and Biomedical Sciences, McMaster University, Hamilton, Ontario, Canada; University of Newcastle upon Tyne, United Kingdom

## Abstract

Heterogeneity is a feature of stem cell populations, resulting from innate cellular hierarchies that govern differentiation capability. How heterogeneity impacts human pluripotent stem cell populations is directly relevant to their efficacious use in regenerative medicine applications. The control of pluripotency is asserted by a core transcription factor network, of which Oct4 is a necessary member. In mouse embryonic stem cells (ESCs), the zinc finger transcription factor Rex1 (Zfp42) closely tracks the undifferentiated state and is capable of segregating Oct4 positive mESCs into metastable populations expressing or lacking Rex1 that are inter-convertible. However, little is currently understood about the extent or function of heterogeneous populations in the human pluripotent compartment. Human ESCs express *REX1* transcripts but the distribution and properties of *REX1* expressing cells have yet to be described. To address these questions, we used gene targeting in human ESCs to insert the fluorescent protein Venus and an antibiotic selection marker under the control of the endogenous *REX1* transcription regulatory elements, generating a sensitive, selectable reporter of pluripotency. *REX1* is co-expressed in OCT4 and TRA-1-60 positive hESCs and rapidly lost upon differentiation. Importantly, *REX1* expression reveals significant heterogeneity within seemingly homogenous populations of OCT4 and TRA-1-60 hESCs. *REX1* expression is extinguished before OCT4 during differentiation, but, in contrast to the mouse, loss of *REX1* expression demarcates a stable, OCT4 positive lineage-primed state in pluripotent hESCs that does not revert back to *REX1* positivity under normal conditions. We show that loss of *REX1* expression correlates with altered patterns of DNA methylation at the *REX1* locus, implying that epigenetic mechanisms may interfere with the metastable phenotype commonly found in murine pluripotency.

## Introduction

Heterogeneity describes mixtures of distinct sub-populations of cells with functional differences that arise due to a balance of stem cell self-renewal and differentiation. In pluripotent stem cells, the cells at the apex of potency make discreet fate decisions, committing to one of numerous, but finite lineage choices, and descend through stages of cellular potential towards differentiated somatic phenotypes. Heterogeneity is a feature of stem cell systems throughout development, including intestinal, neural and hematopoietic stem cells [1], and the fluctuations in gene expression that comprise the heterogeneity in stem cell populations may be a necessary feature, presenting “windows of opportunity”, during which cellular fate choices can be made [1], [2], [3]. The identification and characterization of the cellular hierarchies that distinguish the differentiation capability of cells during development enables control over these processes, permitting the efficient differentiation of cells into tissues suitable for regenerative medicine applications.

In the early mouse embryo, a network of genes, including Oct4, Sox2 and Nanog, establish and maintain the pluripotent state [4], [5], [6], [7], [8]. Pluripotent cells can differentiate into all tissues of the adult organism and represent the highest level of potency from which permanent *in vitro* cell lines, embryonic stem cells (ESCs), have been established. Mouse ESCs closely resemble the “naïve” inner cell mass (ICM) of the blastocyst both in gene expression and differentiation capability [9], [10] but display measurable differences from later mouse epiblast stem cells (EpiSC) [11], [12], [13], which are still considered pluripotent and capable of generating tissues comprising all three germ layers. These observations suggested the existence of a hierarchy within the pluripotent compartment that has recently been explored by several elegant genetic experiments. Mouse ESCs carrying fluorescent reporter proteins under the control of pluripotency-associated transcription factors such as Rex1 [14], Nanog [8] and Stella [15] have described an unappreciated level of heterogeneity present in pluripotent Oct4 expressing ESC cultures. These reports have described the phenomena of metastability within the pluripotent compartment, in which ESCs fluctuate the expression of pluripotent markers as they transit between a naïve and lineage primed state. In particular, expression of the zinc finger transcription factor Rex1 (Zfp42) is exquisitely controlled during early embryogenesis and is sufficient to distinguish cells with an earlier ICM phenotype, capable of re-entering development and contribution in chimeric assays, from cells with later epiblast-like characteristics, that show poor chimeric contribution but good in vitro differentiation [14].

To date, the expression and necessity of genes such as *OCT4*, *SOX2* or *NANOG* have been investigated in undifferentiated hESCs [16], [17], [18] but attempts to explore the presence of a hierarchy within the pluripotent compartment have been limited to extant antibodies to cell surface markers [19], [20].

We previously identified the human *REX1* gene and showed that *REX1* transcripts are expressed in human ESCs and are associated with an undifferentiated phenotype [21]. To gain insight into *REX1* transcript expression, distribution and the nature of pluripotency in hESCs, we used homologous recombination to target the human *REX1* locus with the Venus fluorescent reporter gene [22]. The REX1^Ven/w^ hESC reporter cell lines not only allow a functional enrichment for undifferentiated cells but also describe a subpopulation of *REX1* expressing cells within heterogeneous populations of pluripotent OCT4 or TRA-1-60 expressing hESCs. Fractionation of hESC based on REX1Venus expression reveals a previously hidden hierarchy within the pluripotent compartment, comprising undifferentiated and differentiation primed cells, which lacks the metastability observed in murine ESCs

## Materials and Methods

### Human ESC culture and differentiation

Human ESC line H1 [23] (WiCell) was grown on mitotically-inactivated MEFs in hESC media (Knockout DMEM supplemented with 15% Knockout SR, 1× Non Essential Amino Acids, 1× Glutamax, 1× 2ME (all Invitrogen) and 16 ng/ml bFGF (Peprotech) and passaged with Collagenase type IV (Invitrogen). For antibiotic selection experiments, cells were cultured in hESC media with or without the addition of 1.5 ug/ml puromycin. For monolayer differentiation, hESCs were grown in hESC media on MEF coated 48 well plates and differentiation initiated by substituting the hESC media for DMEM supplemented with 10% FBS, 1× Non Essential Amino Acids and 1× Glutamax. 10 uM retinoic acid (RA) was added to the differentiation media in some experiments. To evaluate hematopoietic differentiation in REX1^Ven/w^ hESCs, EBs were generated by suspension culture methods as previously described [24]. Briefly, undifferentiated REX1^Ven/w^ hESCs were grown on Matrigel to confluence and then treated with Collagenase IV and mechanically scraped off into clumps and incubated overnight in 6-well ultralow attachment plates to allow EB formation (Cornings). For endoderm differentiation of cells isolated using fluorescence activated cell sorting (FACS), cells were grown in hESC media supplemented with Y27632 (Tocris Bioscience) for 24 hrs and then placed in DMEM/F12 media with 1% FBS+100 ng/ml Activin A (Peprotech)+ 100 ng/ml BMP4 for three days. For hematopoietic differentiation, EBs were cultured in StemPro34 serum-free medium (Invitrogen) supplemented with cytokines as follows: 300 ng/ml stem cell factor (SCF; Amgen), 50 ng/ml granulocyte colony stimulating factor (G-CSF; Amgen), 25 ng/ml bone morphogenic protein-4 (BMP-4; R&D systems), 10 ng/ml interleukin-3 (IL-3; R&D systems), 10 ng/ml interleukin-6 (IL-6; R&D systems), and 300 ng/ml Flt-3 ligand (Flt-3 L: R&D systems). The EBs were cultured for 15 days with medium changes every 3 days. For mesoderm differentiation, FACS isolated populations were cultured in hESC media with Y27632 for 24 hrs, followed by a 48 hr treatment with 10 ng/ml BMP4 (Peprotech) and 20 ng/ml bFGF (Peprotech) in DMEM/F12 with 1% NEAA, 2% B27(Invitrogen), 1% ITS(Invitrogen) and 90 uM 2-ME [25].

### Vector construction and homologous recombination (HR)

The REX1-VF2Pu targeting vector was generated by recombineering. Briefly, a SalI/EcoRI cut Venus-F2A-Puro-pA cassette was cloned into SalI/EcoRI cut pL451 (NCI-Frederick) to create pL451+VF2Pu. 50 bp REX1 locus specific homology arms were added to the region spanning Venus to the 3′ Flp site of pL451 by PCR amplification (PrimeStar, Takara) with the REXVenus-F and REXVenus-pL451-R primers (Table S1; primers ordered from Sigma Genosys), producing the REX1VEN PCR product. Bacteria carrying the Human BAC RP11-713C19 and the pSC101-BAD-gba plasmid [26] (containing the Red/ET recombineering genes) were then electroporated with the REXVEN PCR product and correct replacement of the ATG of the *REX1* open reading frame (ORF) within exon 4 by the Venus-F2A-Puromycin cassette was confirmed by PCR and sequencing. REX-Gap-Rep-R and REX-Gap-Rep-F primers were used to add REX 5′ and 3′ specific 50 bp homology arms onto EcoRI/NotI linearised pBS2SK (Stratgene), producing the REXGAP PCR product. Gap repair was performed on the REXVEN BAC with the REXGAP PCR product to generate the pREX1-VF2Pu-TV targeting vector with 2.5 kb 5′ and 4.5 kb 3′ *REX1* specific homology arms, and confirmed by sequencing across HR junctions. The pREX1-VF2Pu-TV plasmid was transferred to the EL250 recombineering strain bacteria (NCI-Frederick) containing an inducible Flp and the FRT flanked PGK-Neo-pA excised.

HESC cell line H1 was pre-treated with 10 uM Y27632 in hESC media for 1 hour and electroporated with 30 ug of AloI linearised pREX1-VF2Pu-TV as previously described [27]. After electroporation, cells were replated on 4DR [28] MEFs in hESC media containing Y27632. 72 hours after electroporation, homologous recombination events were selected for by the addition of 1 ug/ml puromycin for 10 days. Colonies were picked by hand under a dissecting microscope and transferred to MEF coated 4 well plates prior to expansion. Southern blot was performed on 10 ug of PvuII digested hESC genomic DNA with a 470 bp 5′ probe generated by PCR with REXprb-F and REXprb-R, producing an 8.9 kb band from the wild type allele and a 6.8 kb band from the targeted allele.

### Immunofluorescence, high content imaging and analysis

HESCs were cultured in 48 well plates, washed with PBS, fixed with 4% PFA, washed with PBS, permeablised with 100% ice cold methanol and washed with PBS. Cells were stained with Hoechst 33342 and primary antibodies for OCT4 (mouse monoclonal 1∶200, BD #611203), NANOG (rabbit monoclonal 1∶400, Cell Signaling #4903), GATA4 (rabbit polyclonal 1∶300, Sana Cruz #sc-9053) and p21 (rabbit monoclonal 1∶400, Cell Signaling #2947) in 1% BSA in PBS for 2 hours at room temperature or 4°C overnight, washed with PBS and stained with secondary antibodies (Goat anti Mouse AF546 1∶500, Invitrogen # A-11030; Donkey anti Rabbit AF647 1∶500, Invitrogen #A-31573). Analysis was performed as previously described [29], briefly: plates were imaged on a Cellomics ArrayScan HCS reader (Thermo Scientific) or an Operatta High Content Screening System (Perkin Elmer) and images uploaded to a Columbus database (Perkin Elmer) and image analysis of immunofluorescence and reporter fluorescence was performed using Acapella high content and analysis software (Perkin Elmer). Cell nuclei were identified by Hoechst 33342 staining and the fluorescence intensity of the same nuclei in the VENUS, Cy3 and Cy5 channels measured. Custom MatLab (Mathworks) scripts were then used to quantify the fluorescent intensity of each nuclei in all channels and output statistics.

### Flow Cytometry, FACS and CIC assay

HESCs were dissociated to single cells, counted and 2×10^5^ cells co-stained with antibodies (or their corresponding isotype controls) diluted in staining buffer (1% BSA in PBS with 2 mM EDTA) for 30 minutes on ice and then washed 2× with staining buffer. Cells were stained with the viability dye 7 aminoactinomycin D (7-AAD) (Immunotech) to exclude dead cells and analysed on a FACSCalibur or LSRII (BD Biosciences). For FACS, populations were fractionated using an Aria II (BD Biosciences) or a MoFlo (BD). Antibody dilutions were as follows: TRA1-1-60 @ 1∶2000 (AF647 conjugated, BD Biosciences #560122), E-Cadherin @ 1∶100 (PE conjugated ,Santa Cruz #sc-21791-PE), A2B5 @ 1∶100 (APC conjugated, Miltenyi Biotec #130-093-58), CXCR4 @ 1∶100 (APC conjugated, R&D# FAB170A) , CD31-APC @ 1∶100 (BD Pharmingen), CD34-APC @ 1∶100 (Miltenyi Biotech), and CD45-APC @ 1∶100 (Miltenyi Biotech). For the colony initiating cell (CIC) assay, cells were deposited at 25 k and 50 k cells by the ARIA II directly into wells of a 6 well plate containing mitotically inactivated hDFs [30] (hESC derived fibroblasts, 200 k per well) and hESC media and then re-cultured for 12 days. Plates were then fixed with 100% methanol, washed in PBS and stained with OCT4 (mouse monoclonal 1∶200, BD #611203). Plates were imaged using a flatbed scanner (Canon) and colonies enumerated using a custom macro written for ImageJ (rsbweb.nih.gov/ij/).

### mRNA extraction and PCR

mRNA and genomic DNA was isolated from hESCs with a RNA/DNA/Protein Purification kit (Norgenbiotek). mRNA was reverse transcribed into cDNA with an iScript kit (BIORAD), and subject to SYBR Green chemistry based QRT-PCR (Quantitative Real-Time PCR) (GoTaq master mix, Promega). Target genes were quantified relative to the house keeping genes TBP and/or CYCG. The presence of the REX1 targeting vector in genomic DNA was ascertained by gDNA PCR using a common forward primer (REXgDNA-F) in the 5′ UTR of exon 4 combined with either a reverse primer (REXgDNA-R) in the endogenous REX1 ORF (recognizing endogenous REX1; 643 bp band) or a reverse primer (Venus-R) in the Venus ORF (recognizing the REX1-VF2Pu targeting vector; 545 bp band). QRT-PCR primers used in this study are listed in Table S2.

### Bisulphite DNA methylation assay

Genomic DNA was isolated using All-in-one purification kit (Norgen Biotek Corp., Cat #: 24200), and was subjected to bisulfite conversion and treatment as per manufacturer's instructions (EZ DNA Methylation-Gold™ kit, Zymoresearch). Bisulfite converted DNA was PCR amplified using IMMOLASE™ DNA Polymerase (Bioline) cycling at: 95°C for 1 min, [95°C for 30 s, 58°C for 30 s and 72°C for 1 min]×40. Primers used in this study were generated elsewhere [31], [32] and are listed in Table S3. PCR products were cloned into pGEM®-T Easy Vector System I (Promega), purified and sequenced using T7 primer. The sequences were analyzed using QUMA analysis tool (http://quma.cdb.riken.jp/) [33].

### Statistical analysis

Error bars show SEM. Statistical analysis (t-test) was performed with Prism 5 (Graphpad). * = p<0.05, Graphs generated from the automated image analysis are derived from an n of between 3 and 6. Each n involved the analysis of >10,000 cells.

## Results

### 1. Generation of REX1^Ven/w^ human embryonic stem cells

**Figure 1:**
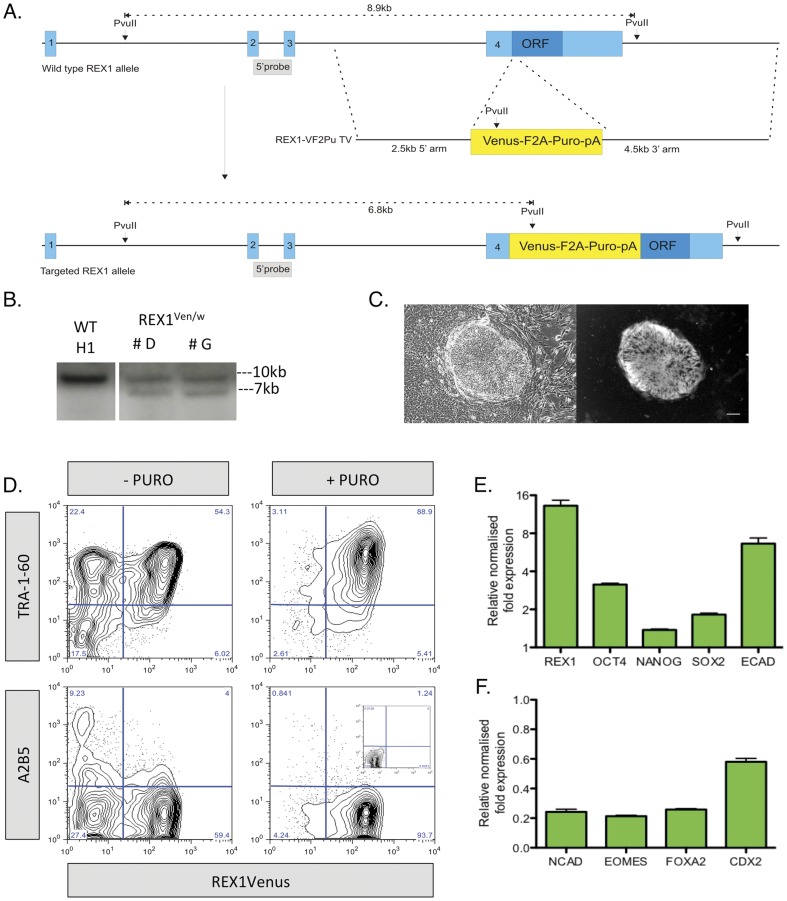
Generation of REX1^Ven/w^ human embryonic stem cells. A) Schematic of the wild type human REX1 allele, targeting vector (REX1-VF2Pu TV) and targeted allele. B) Southern blot confirmation of targeting. C) Phase and fluorescence images of a REX1Ven/w hESCs. Scale bar = 100 microns. D) Flow cytometry on REX1^Ven/w^ cells grown for 7 days in undifferentiated hESC conditions with or without the addition of puromycin co-stained with TRA -1-60 or A2B5. Control inset. E & F) QRT-PCR analysis of pluripotency (E) and differentiation (F) gene transcript expression VEN+ populations isolated by FACS from undifferentiated REX1^Ven/w^ hESCs. All values are normalised relative to the VEN− population = 1.

*REX1* is highly expressed in undifferentiated cultures of hPSC [21], so we used homologous recombination to replace the start codon of an endogenous REX1 allele with a Venus-F2A-puromycin cassette (Fig. 1A) and enriched for HR events by puromycin selection. Two clones with a correctly targeted *REX1* allele (REX1^Ven/w^) were confirmed by southern blotting (Fig. 1B) and displayed bright REX1Venus expression restricted to colonies with an undifferentiated hPSC morphology (Fig. 1C). Karyotyping revealed that the REX1^Ven/w^ clones retained a normal 46 XY chromosomal count (Fig. S1).

### 2. REX1 expression delineates a subpopulation of pluripotent hESCs

REX1Venus expression was confined to a subpopulation of cells co-stained with the human pluripotency-associated cell surface marker TRA-1-60 [34] (Fig. 1D), confirming the association of our *REX1* reporter expression with pluripotency in hPSCs but also establishing that *REX1* displays heterogeneous expression in hPSCs. All REX1Venus-positive (herein referred to as VEN+) cells co-stained with the epithelial marker E-CADHERIN but showed virtually no reactivity with differentiation markers such as A2B5 and CXCR4 (Fig. 1D & Fig. S2A), expressed on ectoderm and endoderm cells, respectively. Functional enrichment for VEN+ cells by the addition of puromycin to REX1^Ven/w^ cultures depleted virtually all spontaneous differentiation (Fig. 1D & Fig. S2B), as measured by loss of A2B5 and CXCR4 expressing cells and TRA-1-60 negative cells, being composed almost uniformly of TRA-1-60 and VEN double positive cells (Fig. 1D). VEN+ cells isolated by FACS display a 13-fold enrichment for *REX1* transcript, when compared to VEN− cells, but less than 3-fold increase in other pluripotency markers such as *OCT4*, *NANOG* and *SOX2* (Fig. 1E), validating the fidelity of the REX1Venus reporter to enrich REX1-expressing cells. Markers of early differentiation, including *N-CAD*, *EOMES*, *FOXA2* and *CDX2* were all enriched in the VEN- cells (Fig. 1F).

### 3. REX1 expression marks a high level domain in the pluripotent hierarchy

**Figure 2:**
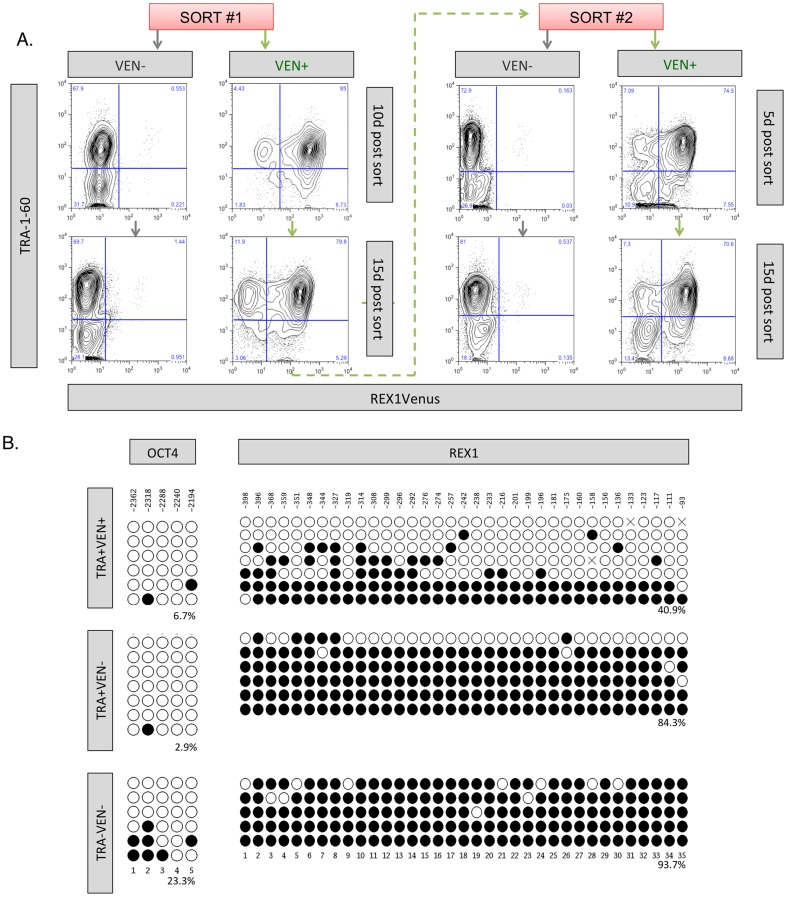
Serial fractionation of REX1^Ven/w^ cultures based upon REX1Venus expression. A) FACS fractionation, re-culture and TRA-1-60 flow analysis of REX1^Ven/w^ hPSCs. B) Bisulphite DNA sequencing on TRA-1-60/REX1Venus hESC populations isolated by FACS for the OCT4 and REX1 promoters. Empty circles designate unmethylated CpG residues and filled circles denote methylated residues. CpG position is provided with reference to transcription start site.

We next tested the hypothesis that *REX1* expressing cells occupy a position towards the top of the pluripotency hierarchy. FACS-fractionated (to 99.9% purity) VEN+ or VEN− populations were separately re-cultured in undifferentiated hPSC conditions and the profile of TRA-1-60 and REX1Venus expression was evaluated over time. Ten days after sorting, the VEN+ fraction contain not only TRA-1-60 expressing VEN+ (TRA+VEN+) cells but had also re-constituted the TRA+VEN− population (Fig. 2A). Cultures derived from the VEN− fraction contained a large proportion of TRA-1-60 positive cells but, in contrast to the behaviour of the VEN+ hPSCs and the murine Rex1-GFP reporter [14], did not re-establish a VEN+ population, even after 2 months of continuous culture (data not shown), despite the demonstrable presence of the REX1-VF2Pu targeting vector in the genomic DNA (Fig. S3). A second serial round of FACS purification performed on the VEN+ cultures derived from the first sort displayed the same pattern of REX1Venus distribution (Fig. 2A), confirming that REX1-expressing VEN+ cells can produce VEN− cells but not the converse, implying that VEN+ cells occupy a higher level in the pluripotent hierarchy than VEN− hPSCs.

The lack of reversion from VEN− to VEN+ cells prompted an investigation of the epigenetic mechanisms that might be regulating the expression of *REX1*. We performed bisulfite genome sequencing on the *REX1* and *OCT4* gene loci on FACS isolated populations demarcated by the expression of both TRA-1-60 and REX1Venus. Assay of the three main populations demonstrated that only the TRA+VEN+ populations displayed hypo-methylation at both the REX1 and OCT4 promoters (Fig. 2B). Therefore, the lack of metastable reversion from VEN− to VEN+ in hESCs could be due to epigenetic changes at the REX1 locus. This suggests that epigenetic modification to the DNA may be responsible for the stability of the VEN− population.

### 4. REX1 expression is rapidly lost upon differentiation

**Figure 3:**
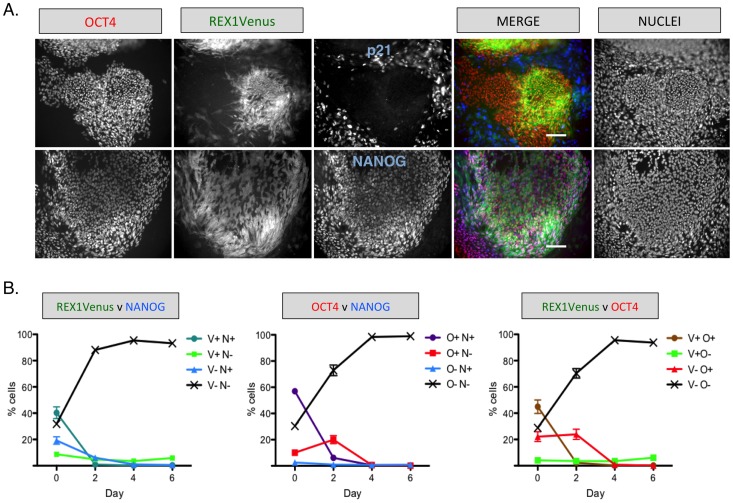
Distribution of pluripotent markers in undifferentiated REX1^Ven/w^ hPSCs. A) Immunocytochemistry for OCT4 (red), NANOG (blue, bottom row) or p21 (blue, top row) in REX1^Ven/w^ cells. Scale bar = 120 microns. B) Quantification of REX1Venus, OCT4 and NANOG expression by high content imaging and automated cell level analysis in undifferentiated cultures (Day 0) and during a time course of retinoic acid induced differentiation (n = 4). V = REX1Venus, O = OCT4, N = NANOG, + = positive, − = negative.

Antibody co-staining of REX1^Ven/w^ hPSCs revealed that both OCT4 and NANOG marked virtually all cells within colonies that were morphology identifiable as undifferentiated, but VEN+ cells were often distributed in a mosaic pattern (Fig. 3A). By contrast, p21, a cell cycle inhibitor associated with the differentiation of hPSCs [35], surrounded the colonies and did not overlap with REX1Venus and OCT4. In addition, differentiation of REX1^Ven/w^ hPSCs in a hematopoietic differentiation assay showed that REX1Venus intensity is not detected in populations of cells that express CD31, CD34 or CD45, all markers of hematopoietic cell lineages [24] (Fig. S4). Like murine Rex1 [14], our data shows that expression of human *REX1* is associated with the pluripotent state and is lost upon differentiation.

**Figure 4:**
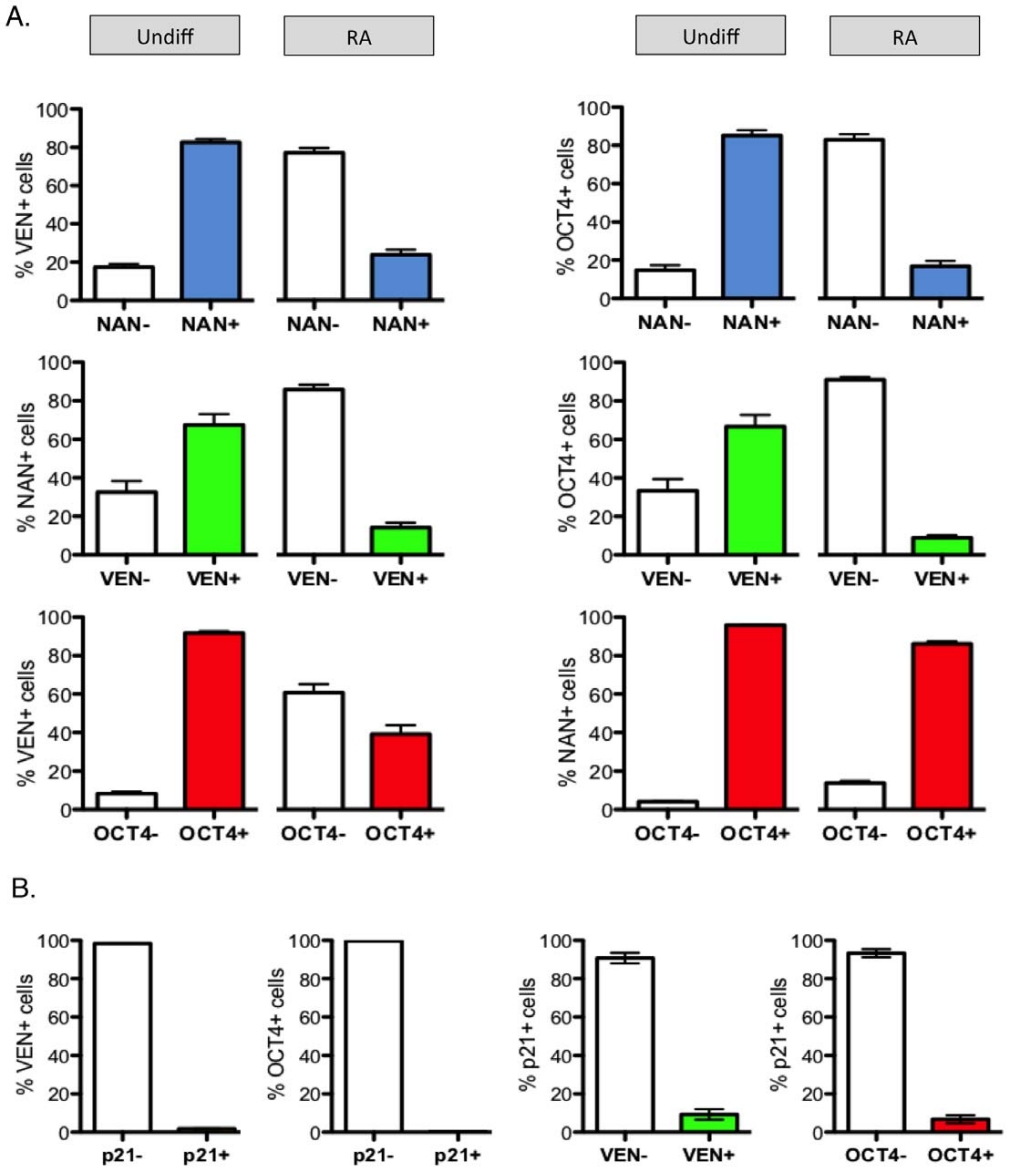
Co-incidence of pluripotency markers in undifferentiated REX1^Ven/w^ hPSC cultures. A) Output of imaging analysis measuring the co-expression of REX1Venus (VEN), OCT4 or NANOG (NAN) pluripotency markers in undifferentiated (Undiff) hESCs and cells treated with retinoic acid (RA) for 2 days, n = 4. B) Output of cell level analysis of p21 co-expression with REX1Venus (VEN) or OCT4 positive cells, n = 4.

We used automated high-content imaging combined with cell annotation software analysis to quantify the overlap of REX1Venus, OCT4 and NANOG expression in undifferentiated and differentiating REX1^Ven/w^ hPSCs. In undifferentiated cultures, just under half of the cells were VEN+ and NANOG+ or VEN+ and OCT4+, with a fifth expressing only NANOG or OCT4 (Fig. 3B). In comparison, over half of undifferentiated hPSCs were NANOG and OCT4 double positive, and fewer were solely OCT4 or NANOG positive. After two days of culturing in conditions that antagonize pluripotency (DMEM + 10% FBS & retinoic acid) [34], REX1Venus and NANOG were virtually absent from REX1^Ven/w^ hPSCs cultures, despite a fifth of the population continuing to express OCT4 (Fig. 3B). Nearly all VEN+ cells were NANOG+ or OCT4+, compared with two thirds of NANOG+ or OCT4+ cells that were VEN+ (Fig. 4A), showing that OCT4 is the most widespread pluripotency marker in hPSC cultures. Upon induction of differentiation, REX1Venus was quickly lost from NANOG+ and OCT4+ cells, and nearly all remaining NANOG-positive cells continued to express OCT4 after 2 days of differentiation (Fig. 4A). Together, these data imply that both REX1 and NANOG mark a subset of cells within the more abundant OCT4-positive population, and that the expression of REX1 and NANOG are extinguished before OCT4 during differentiation. In contrast, the differentiation marker, p21, displayed no appreciable overlap with either OCT4+ or VEN+ cells (Fig. 4B).

### 5. Colony forming capacity is not confined to *REX1* expressing hESCs

**Figure 5:**
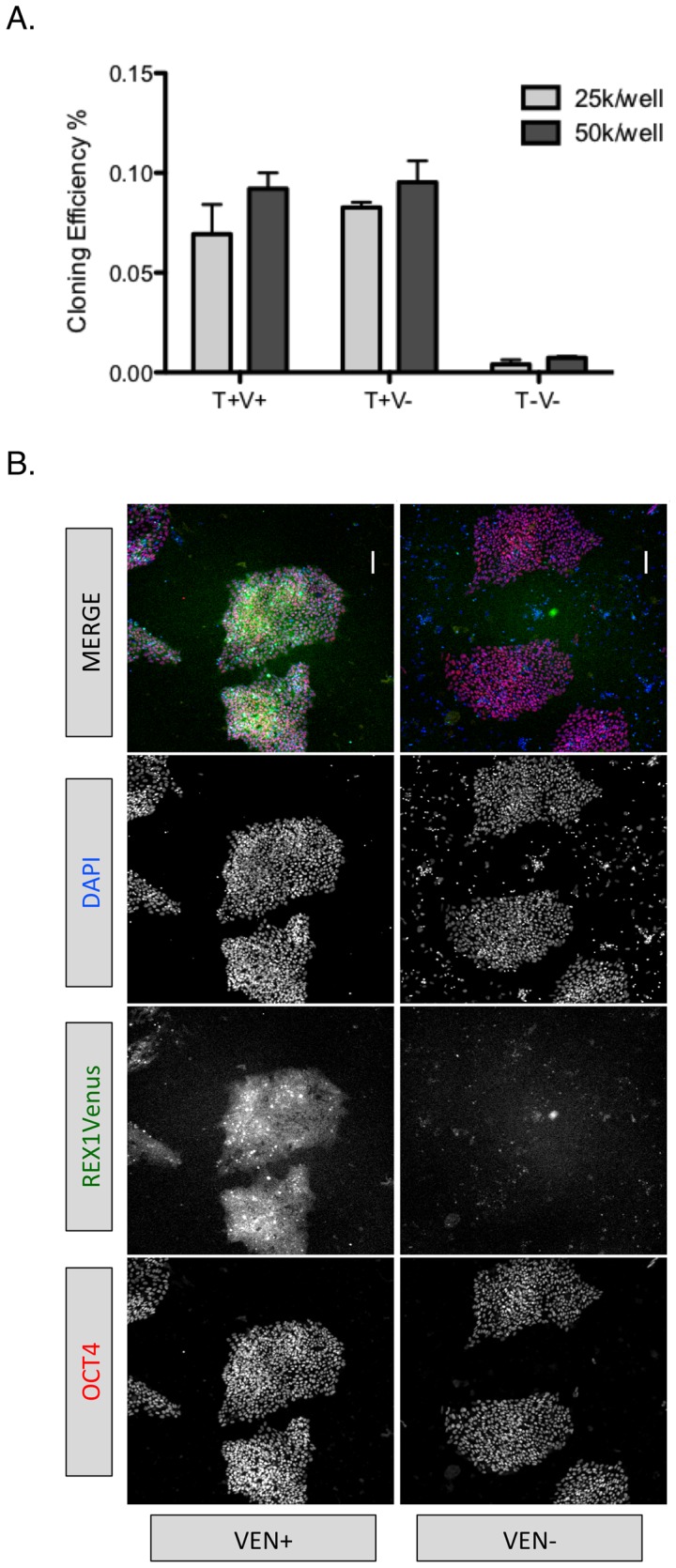
Phenotype of REX1Venus positive and negative populations within REX1^Ven/w^ hESC cultures. A) CIC activity of FACS purified TRA+VEN+ (T+V+), TRA+VEN− (T+V−) and TRA−VEN− (T−V−) populations isolated from undifferentiated cultures of H1 REX1^Ven/w^ cells. B) OCT4 immunocytochemistry and REX1Venus expression in FACS isolated REX1Venus positive (VEN+) or negative (VEN−) populations after 12 days culture. Scale = 120 microns.

A colony initiating cell (CIC) assay, a measure of self-renewal capacity [36], was used to evaluate whether a functional advantage was associated with *REX1* expression (Fig. 5A). The REX1Venus were co-stained with TRA-1-60 and fractionated by FACS into TRA+VEN+, TRA+VEN− and TRA-VEN− populations, seeded back into undifferentiated hESC growth conditions [34] at two defined dilutions and emerging pluripotent colonies quantified by OCT4 expression. The CIC activity of both TRA+VEN+ and TRA+VEN− fractions were comparable, at ∼0.1%, for all dilutions tested, in accordance with the anticipated CIC efficacy for karyotypically normal hPSCs 20,37, and the loss of both REX1Venus and TRA-1-60 marks a differentiated state containing negligible CIC activity. We then analyzed the REX1Venus expression in colonies that emerged from FACS isolated VEN+ or VEN− fractions. The VEN+ derived colonies expressed both REX1Venus and OCT4 protein but the VEN− derived colonies expressed OCT4 and remained uniformly negative for REX1Venus expression (Fig. 5B), a result consistent with our previous findings that VEN− cells are unable to re-establish VEN+ cells (Fig. 2A). These data suggest that the TRA+VEN+ and TRA+VEN− fractions are essentially equivalent in their ability to regenerate OCT4 expressing hESC colonies, and that the higher levels of *REX1* expression associated with the TRA+VEN+ populations are not a requisite for hESC colony formation.

### 6. REX1-negative hPSCs are lineage primed

**Figure 6:**
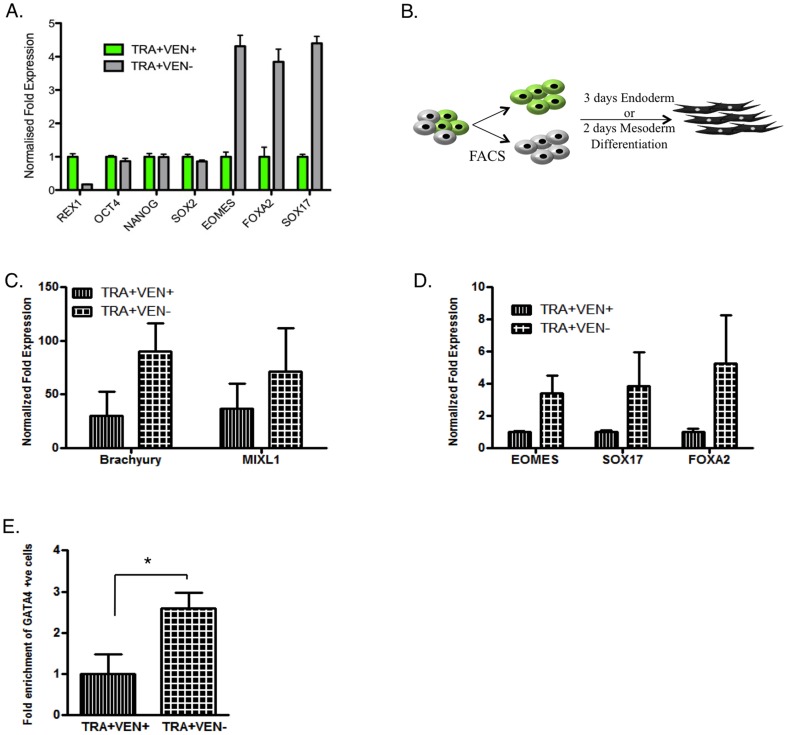
Loss of REX1 within the pluripotent population primes cells for differentiation. **A**) QRT-PCR analysis of gene transcript expression in FACS separated TRA+VEN+ and TRA+VEN− populations. The TRA+VEN− fraction is normalised relative to the TRA+VEN+ population = 1. B) Schematic showing the differentiation treatment of hESCs C & D) QRT-PCR data showing the expression of mesoderm (C) and endoderm (D) lineage associated markers after the TRA+VEN+ and TRA+VEN− fractions were subject to 2 or 3 days of differentiation in mesoderm or endoderm conditions, respectively. C) *BRACHYURY* and *MIXL1* were used as mesoderm associated markers. Undifferentiated TRA+VEN+ population was used as a control. n = 2 D) *EOMES*, *SOX17* and *FOXA2* were used as endoderm specific markers, and gene expression was normalized to TRA+VEN+ day 3 differentiated cells. n = 3 E) Fold enrichment of the percentage of GATA4 positive endoderm cells generated from TRA+VEN− cells relative to those from TRA+VEN+ population after 3 days of treatment with Activin A and BMP4 in low serum media, as observed by immunocytochemistry.

To understand if there was a functional outcome associated with loss of REX1 expression in hPSCs, we then used FACS to isolate the TRA+VEN+ and TRA+VEN− populations and asked whether they had distinct phenotypes. QRT-PCR analysis demonstrated that pluripotency genes such as *OCT4*, *NANOG* and *SOX2* were expressed at equivalent levels in both the TRA+VEN+ and TRA+VEN− populations despite the enrichment for *REX1* transcripts in the TRA+VEN+ population (Fig. 6A). In contrast, the TRA+VEN− population displayed a marked increase in the transcripts of early definitive endoderm specification such as *EOMES* and *SOX17* when compared to the TRA+VEN+ cells (Fig. 6A), as well as the pan or extraembryonic endoderm markers *FOXA2*, *AFP*, *GATA6* and *HNF1B* (Fig. 6A and Fig. S5A). We next tested whether the differential in expression of early lineage marker transcripts between the TRA+VEN+ and TRA+VEN− cells were maintained after guided differentiation in endoderm or mesoderm inducing conditions for 3 or 2 days respectively (Fig. 6B). The TRA+VEN− population displayed a two-fold increase over the TRA+VEN+ population in the expression of mesoderm genes, *BRACHYURY* and *MIXL1*, after 2 days in mesoderm inducing conditions (Fig. 6C). Similarly, the TRA+VEN− cells showed higher transcript expression for the endoderm markers, *EOMES*, *SOX17* and FOXA2 in TRA+VEN− cells after 3 days of differentiation towards the endoderm lineage, when compared to TRA+VEN+ cells (Fig. 6D). The TRA+VEN− cells also generated a higher number of cells expressing GATA4 protein compared to the TRA+VEN+ cells after 3 days of endoderm differentiation (Fig. 6E & Fig. S5B). Similar trends were also evident in an endoderm time course differentiation (Fig. S6) performed on VEN+ cells that were purified by puromycin drug selection or VEN− cells that had been isolated by FACS (Fig. 2A) and then subsequently cultured for several months, implying that the VEN− cells represent a stable lineage primed state.

## Discussion

We have targeted an allele of the transcription factor, *REX1*, in human embryonic stem cells with a fluorescent protein and used this reporter to investigate the pluripotent compartment present in cultures of hESCs. Our *REX1* reporter hESCs have, for the first time, enabled the tracking of *REX1* expression during the culture and differentiation of hESCs. Although it has been understood for some time that undifferentiated hESC cultures often contain cells that have arisen by spontaneous differentiation [34], markers like TRA-1-60 or OCT4 are frequently accepted as faithful markers of seemingly equivalent populations of pluripotent hESCs. Our analysis of REX1^Ven/w^ hESCs revealed that TRA-1-60 and OCT4 expressing cells contain a subpopulation that is demarcated by *REX1* expression, and provides evidence that a similar heterogeneity exists within hESCs that has previously been observed within the undifferentiated murine ESC compartment using a mouse Rex1 ESC reporter line [14]. We have demonstrated the connection between *REX1* and pluripotency by using the puromycin antibiotic selection cassette in our REX1^Ven/w^ hESCs as a mechanism for enriching pluripotent hESCs at the expense of differentiated cells. In addition, we have used prospective isolation of VEN+ cells to annotate the molecular phenotype associated with *REX1* expression. VEN+ cells displayed a comparative enrichment for *REX1* transcripts and lower levels of differentiation markers. However, two distinct subpopulations are evident within TRA-1-60 expressing *REX1* reporter hESCs: TRA+VEN+ and TRA+VEN− cells, with the latter showing similar levels of *OCT4*, *SOX2* and *NANOG* gene transcripts to the TRA+VEN+ but higher levels of early lineage associated markers. In addition, the TRA+VEN− cells showed a greater expression of lineage markers when challenged for differentiation, providing compelling evidence that TRA+VEN− cells are primed for differentiation. Importantly, our finding that distinct subpopulations of hESCs display different aptitudes at differentiation is not without precedent, with others showing that clonal tracking can unmask significant contribution variability in seemingly identical hESCs [38]. Notwithstanding, there are some important caveats to the data we present here. We undertook gene targeting of *REX1* in hESCs principally due to the paucity of commercially available REX1 antibodies that accurately detect this protein. The lack of connection between the *REX1* transcript data that our reporter provides and endogenous REX1 protein levels negates the drawing of conclusions concerning REX1 function in the TRA/VEN populations that we have isolated. In addition, the act of targeting one of the *REX1* alleles could impact the levels of REX1 protein expressed within the hESCs, potentially disturbing the ability of this transcription factor to properly function. The impact of heterozygosity at the Rex1 locus in murine pluripotency is poorly defined, but heterozygous Rex1-GFP reporter murine ESCs continue to participate in the formation of chimeric animals [14], suggesting that any impact on pluripotency appears to be minimal. Interestingly, although Rex1 heterozygous adult mice are viable, the expected Mendelian ratio of their litters is disturbed, a phenotype which has been speculated to occur due to the role of Rex1 in later gametogenesis [39]. With these concerns in mind, knock-in reporter lines continue to provide important insights into biological processes when other conventional reagents are lacking.

Our study features the first in depth investigation of heterogeneity and the stability of sub-populations within the pluripotent compartment of hESC cultures. Using our reporter, we have isolated discrete pluripotent fractions and then mapped the inter conversion between phenotypes. VEN+ cells occupy the top domain of hPSC pluripotency, giving rise to the TRA+VEN− and differentiated TRA-VEN− cells when re-cultured. Unexpectedly, the VEN− cells could only re-generate the TRA+VEN− and TRA-VEN− populations but not the TRA+VEN+, despite the demonstrable presence of the REX1 targeting vector in the gDNA of these cells. To rule out the presence of contaminating wild type hESCs in the TRA+VEN− population we performed serial FACS fractionation and found the same pattern of provenience for the TRA+VEN− cells from the TRA+VEN+ population but not vice versa. The finding that the loss of *REX1* expression, but retention of TRA-1-60, signals commitment to a stable intermediate lineage primed state is in stark contrast to the mouse [14]. Notably, experiments with Nanog, Stella and Rex1 [8], [14], [15] all suggest that murine pluripotent heterogeneity is comprised of dynamic, metastable states [40]; GFP+ cells from each of these reporters can re-establish a GFP− population when isolated and re-cultured and, importantly, vice versa. Why there exists a discrepancy between the human and mouse REX1 reporters remains uncertain, but might describe either the manifestation of distinct growth conditions or species related differences in how early fate allocation is managed. The DNA methylation data presented here suggests a stable epigenetic regulation may govern the irreversible loss of *REX1* expression in hESCs. The metastable nature of other murine pluripotency associated factors, like Stella, is associated with a more plastic regulatory mechanism, which appears to involve histone modifications [15]. Our findings provide some clarity to the ongoing debate concerning the similarity of pluripotent stem cells between mouse and human. Human ESCs share several key features with mESCs that distinguish them from mouse epiblast stem cells (EpiSC), including the expression of two ICM markers, REX1 [21] and KLF4 [41], in combination with a lack of the FGF5 expression, a well-characterised epiblast marker [42]. Whilst hESCs reflect many mESC properties, they do display a growth factor dependency that is more akin to EpiSC than mESC [11], [13], [43]. Human ESCs do not self-renew in response to LIF, as observed in mESCs, possibly due to the lack of diapause in humans [44], [45], [46]. FGF2 and Activin A can maintain undifferentiated cultures of both hESC and mouse EpiSCs [47], strengthening the speculation that hPSCs may represent a later embryonic stage than LIF-dependent mouse ESCs. However, it has been shown that mouse EpiSCs can spontaneously revert to an ES cell-like state when cultured in media containing LIF and serum [48], which is accompanied by a reset of DNA methylation at Rex1 and Stella promoters. It has been demonstrated that FGF-based signaling blocks reversion of mouse EpiSC to an ESC-like state [49], a finding that may help to explain our data showing that the TRA-1-60 and OCT4 positive VEN− hESCs do not convert back to a VEN+ state in normal hESC culture conditions. FGF2 is a common denominator in virtually all hESC media compositions [49], [50], and appears necessary to maintain the long-term self-renewal of hESCs [51], [52], [53]. Thus, we speculate that FGF signaling may play a role in protecting and/or causing DNA methylation of the *REX1* locus in VEN− hESCs and Rex1- EpiSC, implying that the culture conditions that support undifferentiated hESC propagation may limit the metastable gene expression observed in murine ESCs. Recently “naïve” LIF-dependent hESC-like lines have been derived, albeit in an unstable state, that mimic more closely some of the properties of mESCs [54]. Evaluating subpopulations identified by our human *REX1* reporter in the context of “naïve” LIF-dependent hESC-like growth conditions may provide significant insight into how signaling pathways mediate metastability in gene expression.

Why *REX1* transcripts are asymmetrically expressed within the pluripotent hESC compartment is likely linked to the function of *REX1*, of which there is currently a limited understanding. The culture of mESC in signaling conditions that promote a pluripotent ground state yields uniformity of Rex1 expression [36], suggesting that Rex1 is closely associated with the naïve pluripotent state. Rex1 deletion in the mouse embryos and ESCs perturbs both gene expression and differentiation [55], [56], [57]. This phenotype exerted in Rex1-null embryos and ESCs may at least, in part, be due to the role of Rex1 as an epigenetic regulator. Rex1-null blastocysts display hypermethylation of imprinted genes, such as Peg3 and Nespas, and chromatin immunoprecipitation demonstrated that Rex1 binds only to the unmethylated allele of these genes [39]. Rex1 appears to share a common evolutionary ancestor with Ying Yang 1 (Yy1) [58], a ubiquitously expressed zinc finger transcription factor that has a proven role as a mediator of epigenetic regulation [59], including interactions with Polycomb Group (PcG) proteins [58], [60]. PcG proteins are known to repress gene expression by interacting with and changing chromatin structure [61], and are believed to aid in the modulation of PSC fate by inhibition of lineage specific markers [62], with a recent study indicating that Rex1 may also interact with PcGs [63]. More recently, it has been demonstrated that REX1 is an integral part of the mechanism that prevents lyonization in female mouse embryonic stem cells by directly interacting with both the Xist and Tsix loci [64], [65], [66]. A conserved function for *REX1* in human X-inactivation remains to be discovered, although it has been questioned whether X-inactivation via the governance of *XIST* expression by *TSIX* even occurs in human cells [67], [68]. Notwithstanding, the hESCs targeted in this study are male, and only recently have advances permitting the derivation of female hESCs with two active X chromosomes [54], [69], affording an opportunity to explore REX1 function in this area. Significantly, LIF-based signaling appears to play a strong role in the generation of human induced pluripotent stem cells (iPSC) that contain two active X chromosomes [70], providing an intriguing model for exploring REX1 function in human lyonization.

It is clear that identifying and characterising the subpopulations that occur within hESC culture heterogeneity can yield significant increases in our understanding of these important cells and have direct impact on their future utility in drug discovery and therapeutic applications. The *REX1* reporter lines described here represent a powerful new tool for understanding human pluripotency.

## Supporting Information

Figure S1 Normal 46XY karyotype, assayed by WiCell Institute, of two H1 subclones expressing REX1-VF2Pu targeting vector.
(TIF)

Figure S2 A) Flow cytometric analysis of REX1^Ven/w^ cells grown for 7days in undifferentiated hESC conditions with or without puromycin co-stained with E-CADHERIN (E-CAD) or CXCR4. B) Histograms of REX1Venus expression with (green line) or without (blue line) 7 day puromycin treatment. Control H1 hESCs (red line).
(TIF)

Figure S3 PCR on genomic DNA for the presence of the REX1-VF2Pu targeting vector (REX1 TV) versus control endogenous REX1 locus (REX1 END). Samples assayed: Wild type H1 hESC (H1 wt), TRA-1-60/REX1Venus fractions (TRA VEN) and VEN− cultures after 7 passages (RXVen−).
(TIF)

Figure S4 Hematopoietic differentiation of REX1^Ven/w^ cells. REX1 reporter cells were differentiated in embryoid bodies in conditions that induce blood formation and assayed at day 4, 10 and 15 for A) REX1Venus expression and B) markers of hematopoietic specification CD31, CD34 and CD45.
(TIF)

Figure S5 A) QRT-PCR of undifferentiated FACS isolated TRA+VEN+ and TRA+VEN− cells for extraembryonic endoderm markers. Gene expression is normalized to the housekeeping gene *TBP*, and is relative to TRA+VEN+ fraction (n = 2) B) Cells were isolated by FACS, re-seeded and the next day treated with endoderm-inducing conditions for 3 days before fixation and staining with GATA4 and OCT4. Scale = 120 microns.
(TIF)

Figure S6 QRT-PCR of several endoderm markers, *SOX17*, *EOMES*, *FOXA2*, *Goosecoid (GSC)*, *Cerberus-like (CER)* and *GATA4*, over a three day (d0-d3) time-course analysis of puromycin selected VEN+ (dashed-line) cells, and VEN− (solid-line) cells (n = 1). Single cells were seeded in Y27632 for 24 hrs (d = 0) before treating for endoderm differentiation for three days. Gene expression is normalized to housekeeping gene *TBP*, and is relative to d0 VEN− control; n = 1.
(TIF)

Table S1 Recombineering primers used to generate the pREX1-VF2Pu-TV targeting vector.
(PDF)

Table S2 QRT-PCR primers used in the study
(PDF)

Table S3 *REX1* and *OCT4* primers for amplifying bisulfite converted gDNA for DNA methylation analysis.
(PDF)
