# Supplementary figures and images for: Demarcation of Stable Subpopulations within the Pluripotent hESC Compartment
Source: PLoS One. 2013 Feb 21;8(2):e57276. doi: 10.1371/journal.pone.0057276 (PMC3578859; doi:10.1371/journal.pone.0057276)

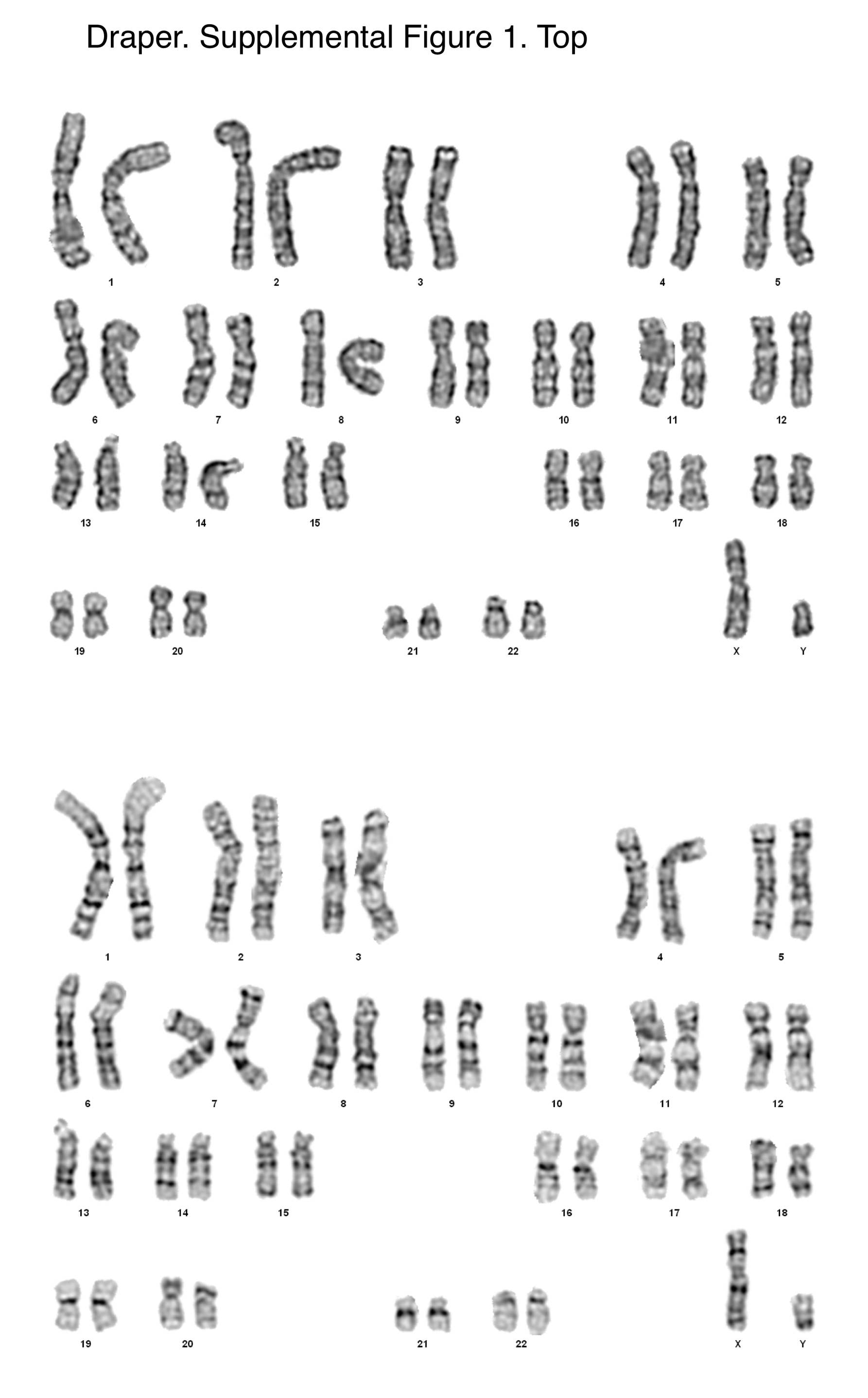

Supplement: Figure S1 — Normal 46XY karyotype, assayed by WiCell Institute, of two H1 subclones expressing REX1-VF2Pu targeting vector. (TIF) [file pone.0057276.s001.tif]

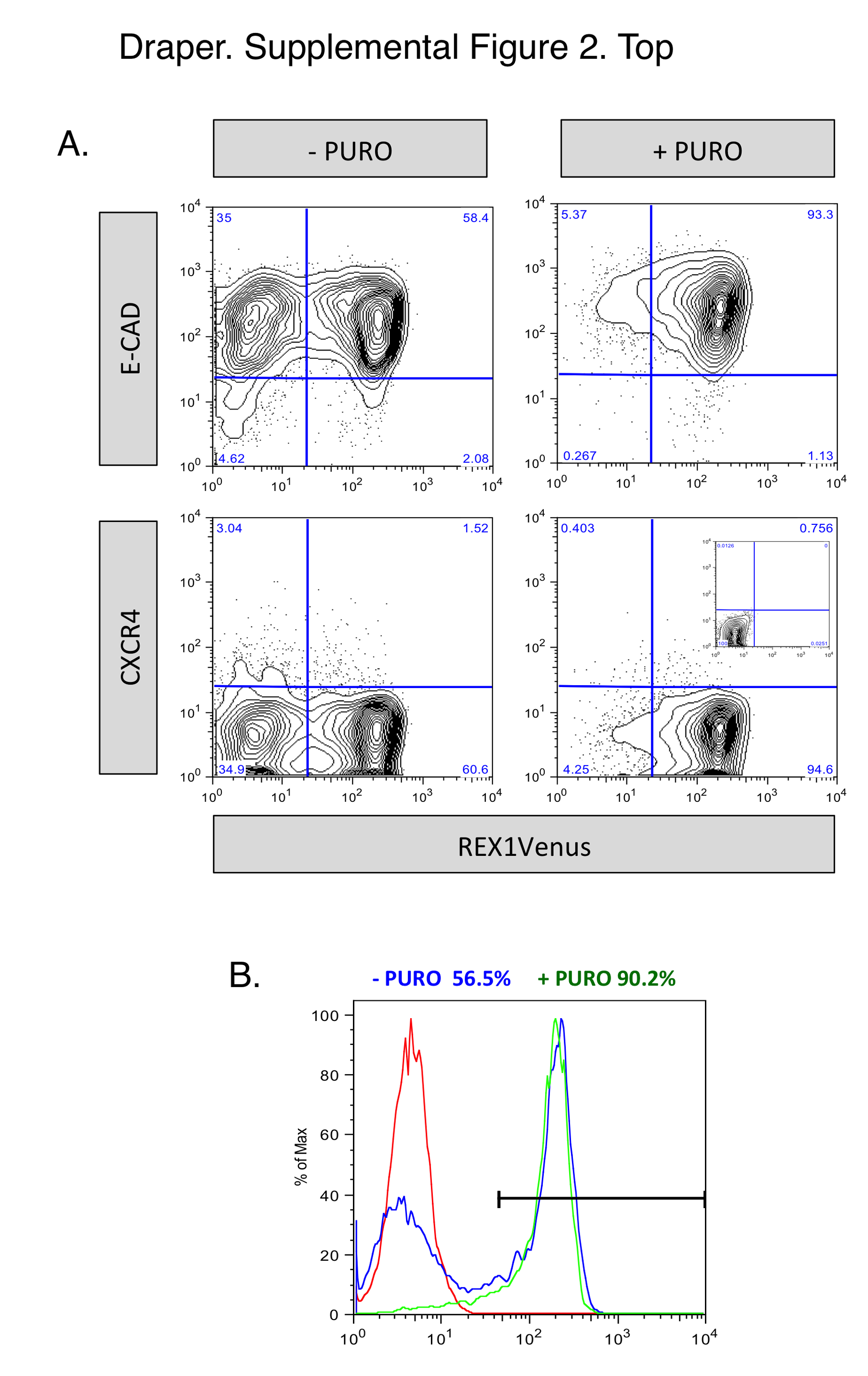

Supplement: Figure S2 — A) Flow cytometric analysis of REX1Ven/w cells grown for 7days in undifferentiated hESC conditions with or without puromycin co-stained with E-CADHERIN (E-CAD) or CXCR4. B) Histograms of REX1Venus expression with (green line) or without (blue line) 7 day puromycin treatment. Control H1 hESCs (red line). (TIF) [file pone.0057276.s002.tif]

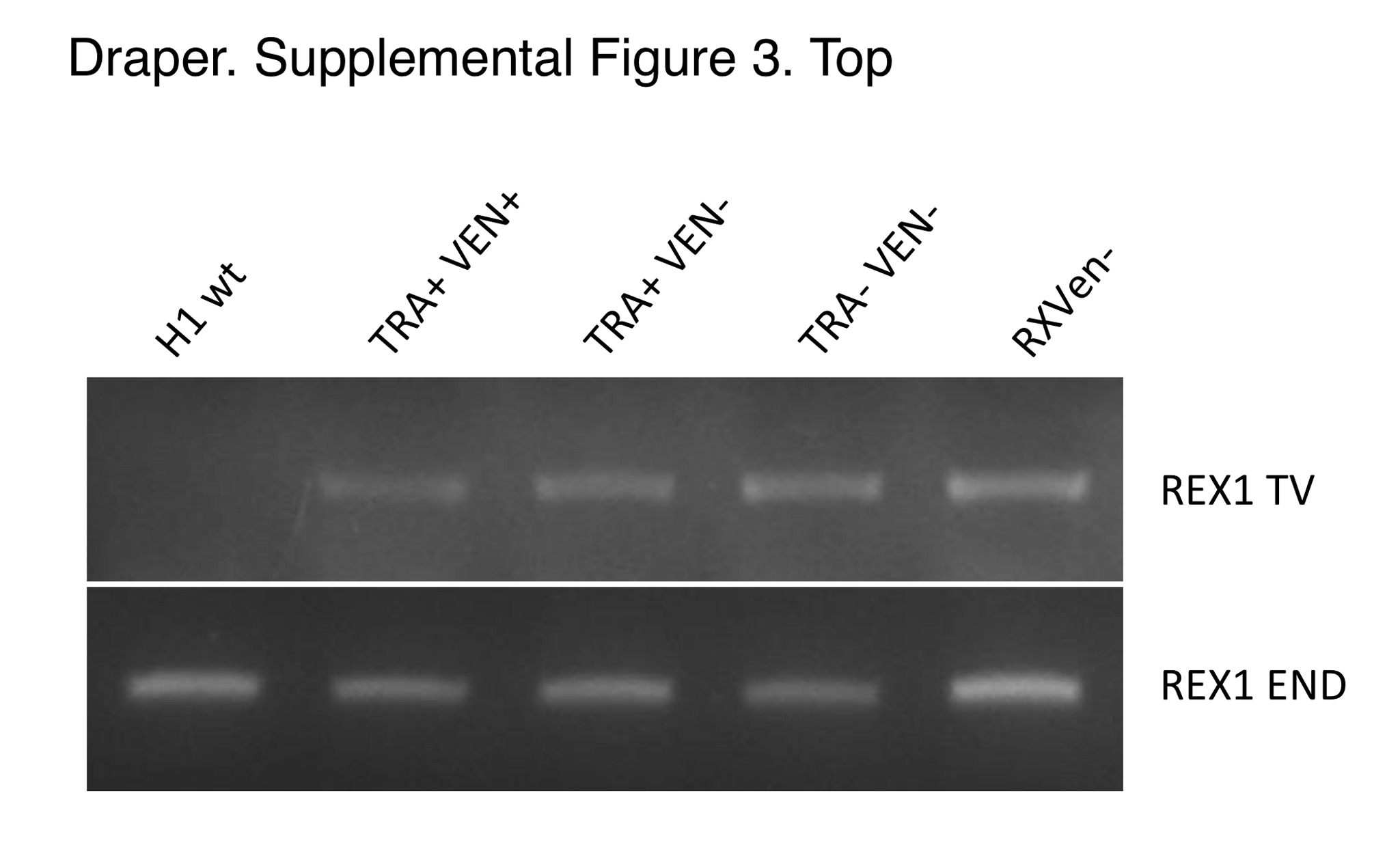

Supplement: Figure S3 — PCR on genomic DNA for the presence of the REX1-VF2Pu targeting vector (REX1 TV) versus control endogenous REX1 locus (REX1 END). Samples assayed: Wild type H1 hESC (H1 wt), TRA-1-60/REX1Venus fractions (TRA VEN) and VEN− cultures after 7 passages (RXVen−). (TIF) [file pone.0057276.s003.tif]

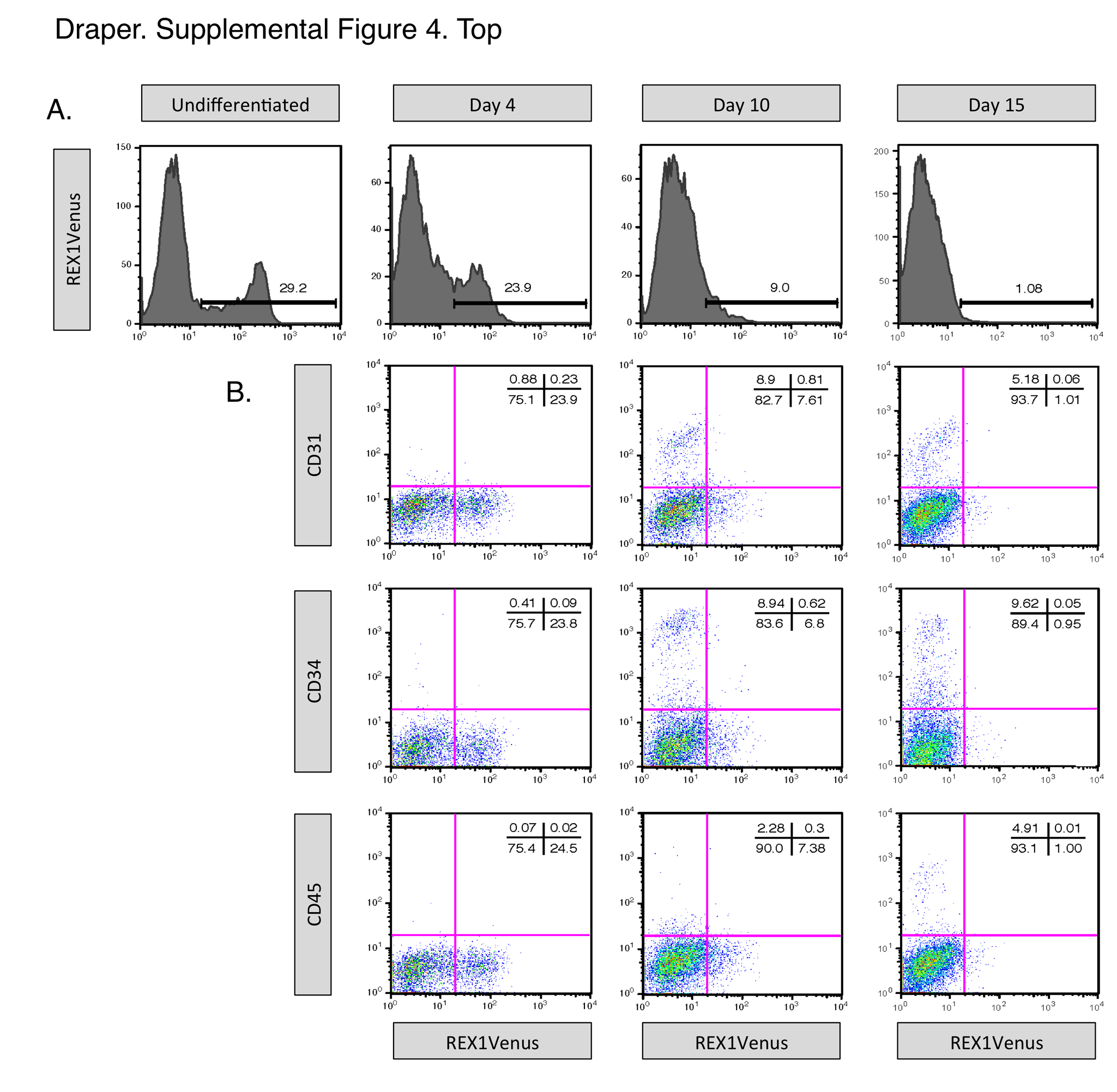

Supplement: Figure S4 — Hematopoietic differentiation of REX1Ven/w cells. REX1 reporter cells were differentiated in embryoid bodies in conditions that induce blood formation and assayed at day 4, 10 and 15 for A) REX1Venus expression and B) markers of hematopoietic specification CD31, CD34 and CD45. (TIF) [file pone.0057276.s004.tif]

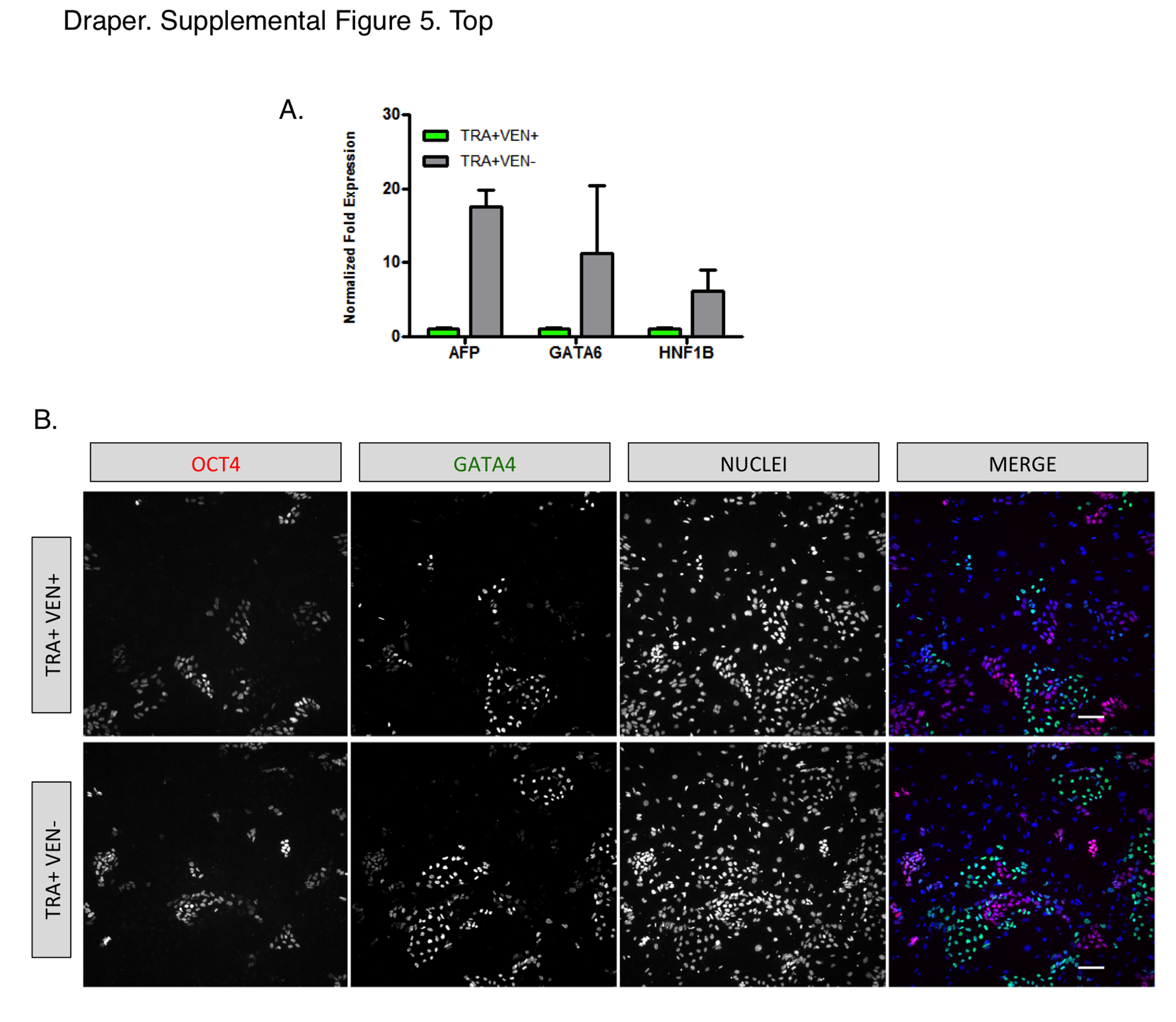

Supplement: Figure S5 — A) QRT-PCR of undifferentiated FACS isolated TRA+VEN+ and TRA+VEN− cells for extraembryonic endoderm markers. Gene expression is normalized to the housekeeping gene TBP, and is relative to TRA+VEN+ fraction (n = 2) B) Cells were isolated by FACS, re-seeded and the next day treated with endoderm-inducing conditions for 3 days before fixation and staining with GATA4 and OCT4. Scale = 120 microns. (TIF) [file pone.0057276.s005.tif]

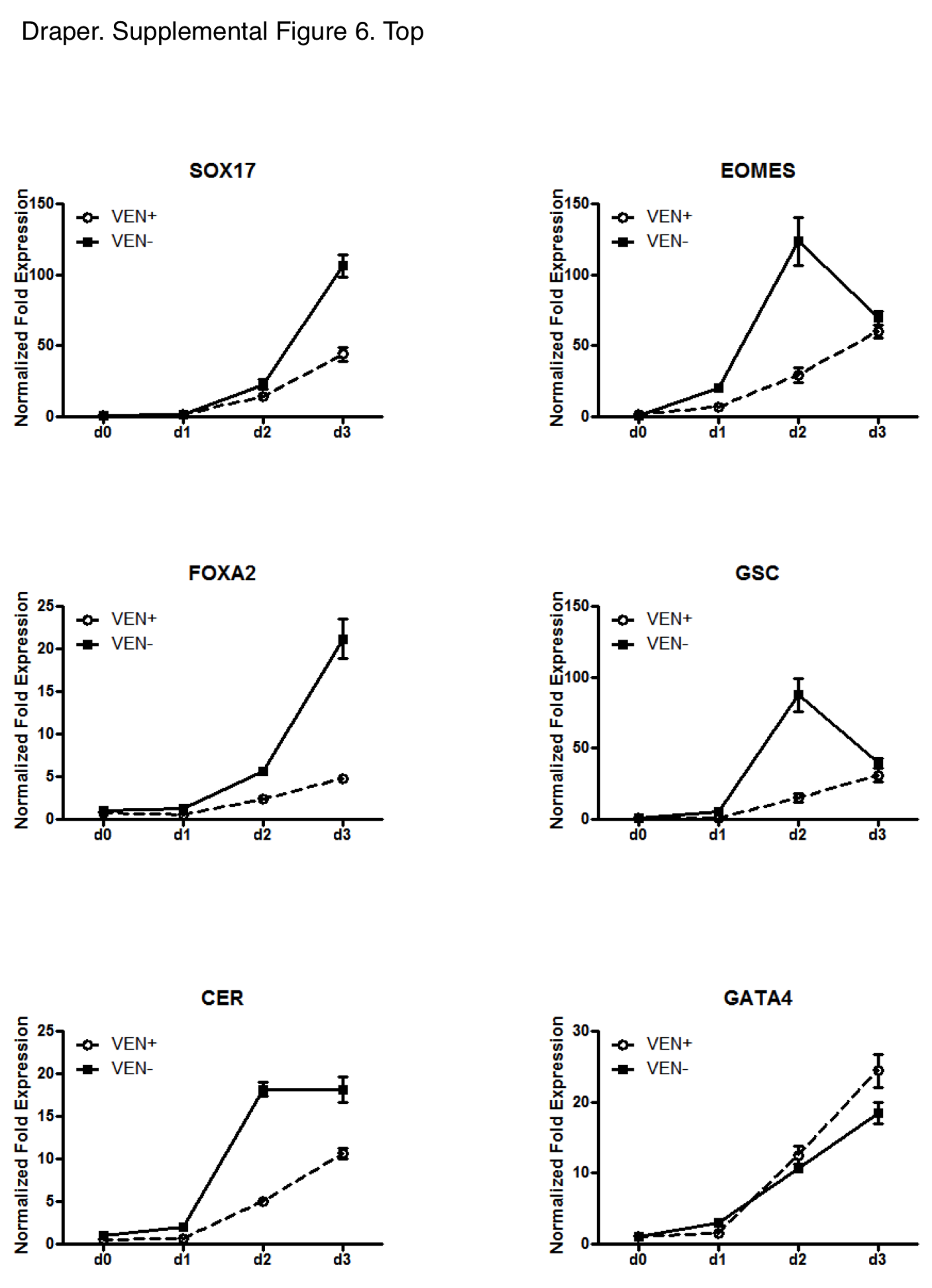

Supplement: Figure S6 — QRT-PCR of several endoderm markers, SOX17, EOMES, FOXA2, Goosecoid (GSC), Cerberus-like (CER) and GATA4, over a three day (d0-d3) time-course analysis of puromycin selected VEN+ (dashed-line) cells, and VEN− (solid-line) cells (n = 1). Single cells were seeded in Y27632 for 24 hrs (d = 0) before treating for endoderm differentiation for three days. Gene expression is normalized to housekeeping gene TBP, and is relative to d0 VEN− control; n = 1. (TIF) [file pone.0057276.s006.tif]
