# Supplementary material for: Demarcation of Stable Subpopulations within the Pluripotent hESC Compartment
Source: PLoS One. 2013 Feb 21;8(2):e57276. doi: 10.1371/journal.pone.0057276 (PMC3578859; doi:10.1371/journal.pone.0057276)
Supplement: Table S2 — QRT-PCR primers used in the study (PDF) [file pone.0057276.s008.pdf]

Table 2. Q-RT-PCR primers

Gene	Forward primer 5'-3'	Reverse primer 5'-3'
REX1	GCCTTCACTCTAGTAGTGCTCACAGT	GGCAGTAGTGATCTGAGTAAGCTGTCT
OCT4	TGGGCTCGAGAAGGATGTG	GCATAGTCGCTGCTTGATCG
NANOG	TGATTTGTGGGCCTGAAGAAA	GAGGCATCTCAGCAGAAGACA
SOX2	TACAGCATGTCCTACTCGCAG	GAGGAAGAGGTAACCACAGGG
ECAD	AGGAATTCTTGCTTTGCTAATTCTG	CGAAGAAACAGCAAGAGCAGC
NCAD	CCCACACCCTGGAGACATTG	GCCGCTTTAAGGCCCTCA
EOMES	CGGCCTCTGTGGCTCAAA	AAGGAAACATGCGCCTGC
FOXA2	GGGAGCGGTGAAGATGGA	TCATGTTGCTCACGGAGGAGTA
CDX2	CTGGAGCTGGAGAAGGAGTTTC	ATTTTAACCTGCCTCTCAGAGAGC
SOX17	GGCGCAGCAGAATCCAGA	CCACGACTTGCCCAGCAT
BRACHYURY	TGCTTCCCTGAGACCCAGTT	GATCACTTCTTTCCTTTGCATCAAG
MIXL1	AAGCCCCAGCTGCCTGTT	CCCTCCAACCCCGTTTG
AFP	TGGGACCCGAACTTTCCA	GGCCACATCCAGGACTAGTTTC
HNF1B	TCACAGATACCAGCAGCATCAGT	GGGCATCCCAGGCTTGTA
GATA6	GCGGGCTCTACAGCAAGATG	ACAGTTGGCACAGGACAATCC
GATA4	TCCAAACCAGAAAACGGAAGC	GCCCGTAGTGAGATGACAGG
CER	ACAGTGCCCTTCAGCCAGACT	ACAACTACTTTTTCACAGCCTTCGT
GSC	GAGGAGAAAGTGGAGGTCTGGTT	CTCTGATGAGGACCGCTTCTG
