# Supplementary material for: Demarcation of Stable Subpopulations within the Pluripotent hESC Compartment
Source: PLoS One. 2013 Feb 21;8(2):e57276. doi: 10.1371/journal.pone.0057276 (PMC3578859; doi:10.1371/journal.pone.0057276)
Supplement: Table S3 — REX1 and OCT4 primers for amplifying bisulfite converted gDNA for DNA methylation analysis. (PDF) [file pone.0057276.s009.pdf]

Table 3. Bisulphite DNA methylation sequencing primers

Gene	Forward primer 5'-3'	Reverse primer 5'-3'
REX1	GGTTTAAAAGGGTAAATGTGATTATATTTA	CAAACTACAACCACCCATCAAC
OCT4	ATTTGTTTTTTGGGTAGTTAAAGGT	CCAACTATCTTCATCTTAATAACATCC
